# Trends in perivascular macrophages research from 1997 to 2021: A bibliometric analysis

**DOI:** 10.1111/cns.14034

**Published:** 2022-12-13

**Authors:** Lv Xie, Li Zheng, Weijie Chen, Xiaozhu Zhai, Yunlu Guo, Yueman Zhang, Yan Li, Weifeng Yu, Zhongmeng Lai, Ziyu Zhu, Peiying Li

**Affiliations:** ^1^ Department of Anesthesiology Clinical Research Center, Renji Hospital, Shanghai Jiao Tong University School of Medicine Shanghai China; ^2^ Department of Anesthesiology Fujian Medical University Union Hospital Fuzhou Fujian China

**Keywords:** bibliometric analysis, brain, obesity, perivascular adipose tissue, perivascular macrophages

## Abstract

**Introduction:**

Perivascular macrophages (PVMs) play pivotal roles in maintaining the physiological function of the brain. Dysfunction of PVMs is emerging as an important mechanism in various disease conditions in the brain.

**Methods:**

In this work, we analyzed recent research advances in PVMs, especially in the brain, from the Web of Science (WoS) core database using bibliometric analysis based on the search terms “perivascular macrophages” and “perivascular macrophage” on October 27, 2021. Visualization and collaboration analysis were performed by Citespace (5.8 R3 mac).

**Results:**

We found 2384 articles published between 1997 and 2021 in the field of PVMs, which were selected for analysis. PVMs were involved in several physio‐pathological fields, in which Neurosciences and Neurology, Neuroscience, Immunology, Pathology, and Cardiovascular System and Cardiology were most reported. The research focuses on PVMs mainly in the central nervous system (CNS), inflammation, macrophage or T‐cell, and disease, and highlights the related basic research regarding its activation, oxidative stress, angiotensin II, and insulin resistance. Tumor‐associated macrophage, obesity, myeloid cell, and inflammation were relatively recent highlight keywords that attracted increasing attention in recent years. Harvard Univ, Vrije Univ Amsterdam, occupied important positions in the research field of PVMs. Meanwhile, PVM research in China (Peking Univ, Sun Yat Sen Univ, Shanghai Jiao Tong Univ, and Shandong Univ) is on the rise. Cluster co‐citation analysis revealed that the mechanisms of CNS PVMs and related brain diseases are major specialties associated with PVMs, while PVMs in perivascular adipose tissue and vascular diseases or obesity are another big category of PVMs hotspots.

**Conclusion:**

In conclusion, the research on PVMs continues to deepen, and the hotspots are constantly changing. Future studies of PVMs could have multiple disciplines intersecting.

## Graphical abstract

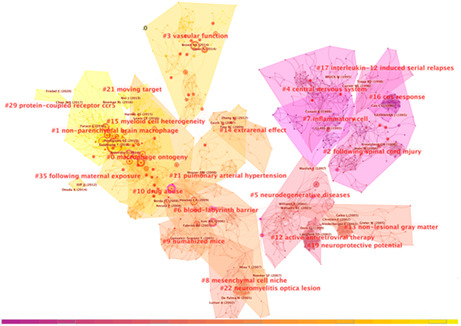

## 1 INTRODUCTION

Perivascular macrophages (PVMs) are located in all organs of our body, and their functions vary from body parts and local environments. Many studies have focused on tissue‐resident macrophage origination and maintenance.^1^, ^2^, ^3^ In recent years, there have been many related studies on PVMs in different tissues and different subgroups, including tumors, uterus, lungs, and adipose tissues, there are also some research and clinical trials on targeted drug therapy of PVMs in peripheral tissues.^4^ Perivascular adipose tissue is the special category of PVMs that participate in the inflammation process and affect cardiovascular disorders including atherosclerosis, obesity, hypertension, diabetes,^5^, ^6^, ^7^, ^8^, ^9^ and atrial fibrillation.^10^ PVMs also take part in the pathological process of tumors.^11^, ^12^ Turnsek TL and Hatlen RR have recently proposed the relationship between perivascular niche in the central nervous system (CNS) and tumor microenvironment (TME) of glioblastoma.^13^, ^14^

The dual roles of PVMs in immune‐to‐brain signaling have aroused great interest in research in this regard.^15^ Previous studies have shown that macrophages have M1/M2 classification, and the clinical impact of M1/kill and M2/repair responses plays pivotal roles in many diseases including infections, cancer, autoimmunity, and atherosclerosis.^16^, ^17^, ^18^ PVMs have been confirmed to present M1 proinflammatory polarization, and the presence or shifting of PVMs subpopulations after ischemic injury was also observed.^19^ Evidence shows that subpopulations of PVMs participated in “tumor microenvironment of metastasis” (TMEM).^20^ However, recent studies revealed that PVMs M2 type might have both protective and destructive effects, and PVMs M2 type expressed in both normal or tumor tissue,^21^ and due to the complex nature of PVMs, it is also difficult to define the status of PVMs as M1/M2.^22^ Thus, the exact role and classification of PVMs warrant further in‐depth studies.

Brain perivascular cells are a large class of immunoregulatory cells that connect the central nervous system (CNS) with the peripheral immune system which contain microglia, monocytes/macrophages, pericytes, and brain dendritic cells.^23^, ^24^ Perivascular cells exist in Virchow–Robin space which was a hot topic around 2000.^25^, ^26^, ^27^ As one type of perivascular cells, PVMs take part in many brain diseases, such as Alzheimer's disease, stroke,^28^ HIV/SIV dementia or encephalopathy, and Parkinson's disease.^29^, ^30^ PVMs in the CNS appear to be very similar to those blood‐derived macrophages.^31^ Research into the inflammatory mechanisms, involving macrophages, granulocytes, T‐cells, B‐cells, and activated microglia, and the pathogenic role of PVMs in brain diseases and many diseases mentioned above may serve as targets for therapeutic intervention.

It is of great importance and interest to look into the distribution and progress of research in the PVMs field. In this study, we used Citespace, bibliometric analysis and clusters as unit, especially selecting larger or newer typical clusters for analysis, a key foothold of the Citespace co‐citation analysis; we performed a comprehensive analysis of knowledge structure, evolution process, and important references in the field of PVMs through macro‐ to micro‐angle, and from overall to partial aspect and demonstrated that the research of PVMs continues to deepen, and the hotspots are constantly changing.

## 2 METHODS

We performed a bibliometric analysis on October 27, 2021, by using the Web of Science (WoS) core database using the search terms “perivascular macrophages” and “perivascular macrophage” in the title/abstract/keywords between 1950 and 2021. The total citation number and average number of citations for each report of all results were collected in the WoS analysis tool. Data were extracted from the WoS database to identify collaborations among categories, keywords, institutions, and references using Citespace (5.8.R3 mac).

Citespace co‐occurrence analysis was used to identify the most important category and institution. Each node represents one category or institution and the size of the node reflects its frequency. The category labels are exhibited beside the node. Centrality is counted and shown as “purple circle” that connects several articles and plays a pivot role in the network. The “red circle” means the category or institution was or is still a hotspot. We used the minimum spanning tree pruning method to make the keyword co‐occurrence analysis network simple and beautiful. Each node represents one keyword. The frequently used keywords are exhibited beside the node.

Reference cocitation analysis network was shown in the cluster view and timeline view. Modularity and average silhouette score were counted for judging the effect of cluster mapping. Modularity means the data distribution pattern has passed the standard quality control, while modularity >0.3 represents a significant cluster structure. The average silhouette score combines cohesion and separation together and examines whether each cluster has enough similarity, silhouette score >0.5 meaning reasonable clustering, and a silhouette score >0.7 convincing clustering in Citespace. In cluster view, the labels of each cluster were displayed alongside the blocks. In the timeline view, cluster labels appeared on the right‐hand side. The hue of the clusters indicates the timing of the cocitation linkages. The red hue denotes a somewhat early citation year, whereas the yellow color indicates a very recent citation time

## 3 RESULTS

### 3.1 Preliminary citation statistics analysis of 2384 articles published between 1997 and 2021

We found 2384 articles in the initial search of the WoS database under the search terms “perivascular macrophages” and “perivascular macrophage.” Of these papers, 93,895 reports had cited these articles, with a total citation number of 120,854. The average number of citations for each report was 50.69.

**FIGURE 1:**
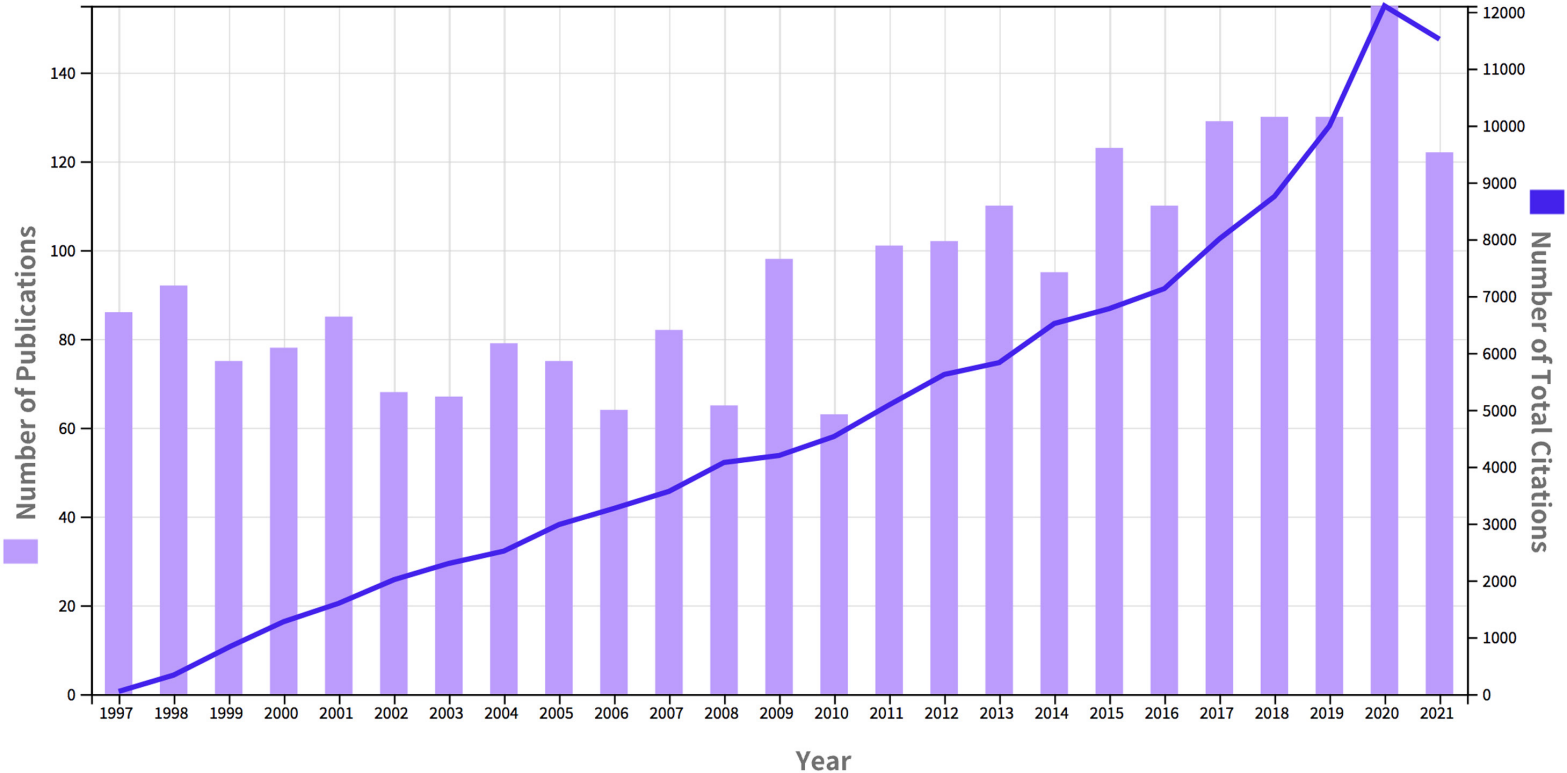
Annual number of publications and annual number of total citations in perivascular macrophages from 1997 to 2021 in Web of Science.

Articles published during 1997–2009 had relatively stable quantities between 60 and 100. Year 2010 had 63 articles which were lowest in those years. Articles grew steadily with the most in 2020 which had 155 articles published. Citation numbers had steady growth from 1997 (*n* = 46) to 2020 (*n* = 12,104). See Figure 1.

### 3.2 Citespace category co‐occurrence analysis revealed pathology and immunology as dominant categories

**FIGURE 2:**
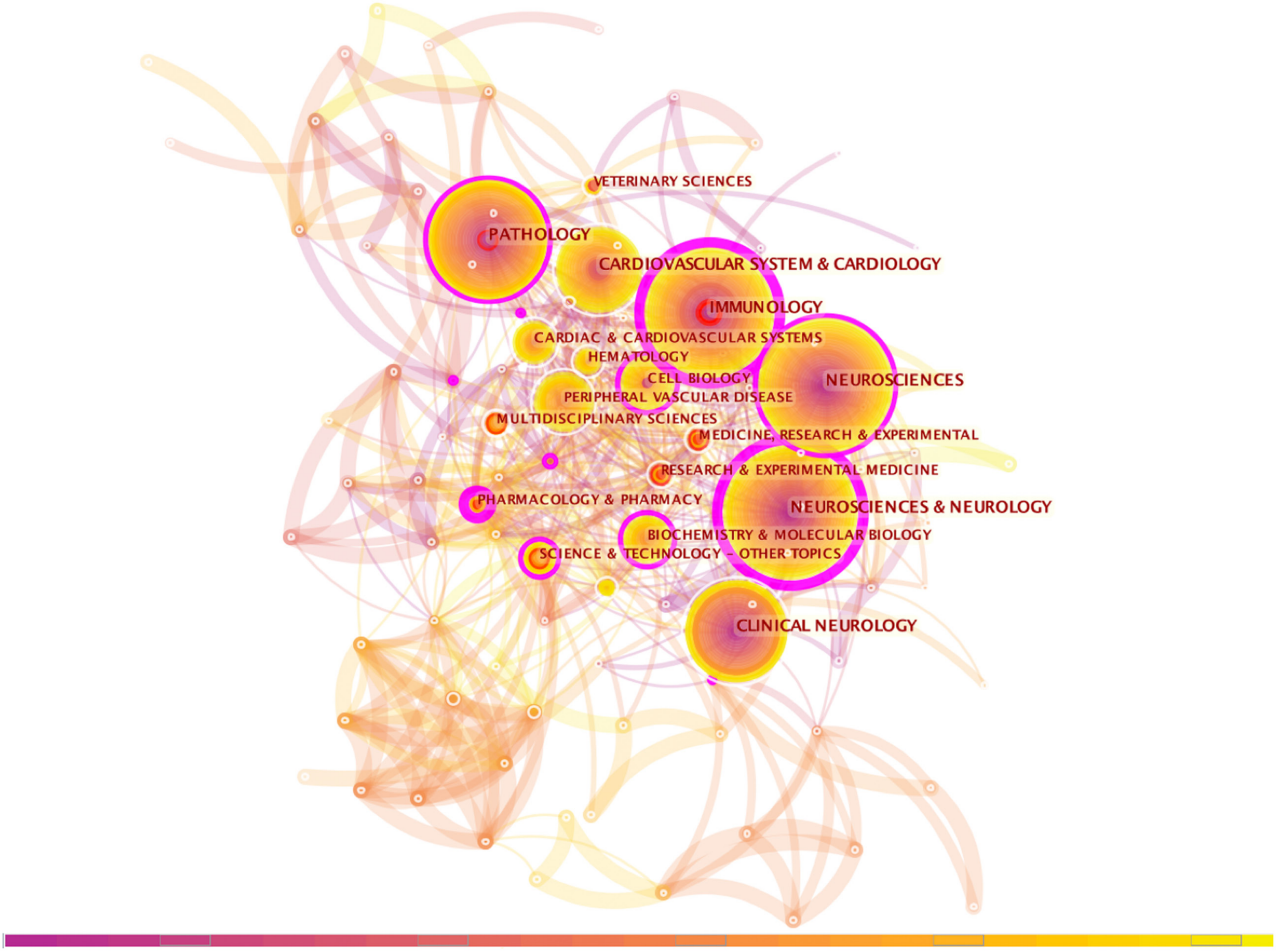
Citespace category co‐occurrence analysis network in perivascular macrophages from 1997 to 2021. Each node represents one category and the size of the node reflects its frequency. The category labels are exhibited beside the node. Centrality is counted and shown as “purple circle” that connect several articles and play a pivot role in the network. The “red circle” means the category was or is still a hot spot.

Citespace category co‐occurrence analysis network is shown in Figure 2. Top 10 categories by frequency, burst, and sigma are shown in Table S1. Burst value reflects the rise and fall of research in the field; the larger the burst value is, the more it can show the cutting‐edge situation in this field. Sigma value is an index that reflects both centrality and burst which is calculated based on these two values, nodes with higher centrality, and burst have higher sigma values, indicating both structural and citational importance. Neurosciences and Neurology, Neuroscience, Immunology, Pathology, and Cardiovascular System and Cardiology were the top five categories by frequency. Among these “biggest circles,” Pathology had the top burst value (18.97) and top sigma value (8.91). Immunology had both “purple circle” and “red circle.” Science and Technology, Surgery, and Research and Experimental Medicine have emerged as hot subjects with both high burst and sigma values.

### 3.3 Keyword co‐occurrence analysis revealed “central nervous system,” “inflammation,” “T cell,” and “expression” as the leading keywords in the PVMs research field

**FIGURE 3:**
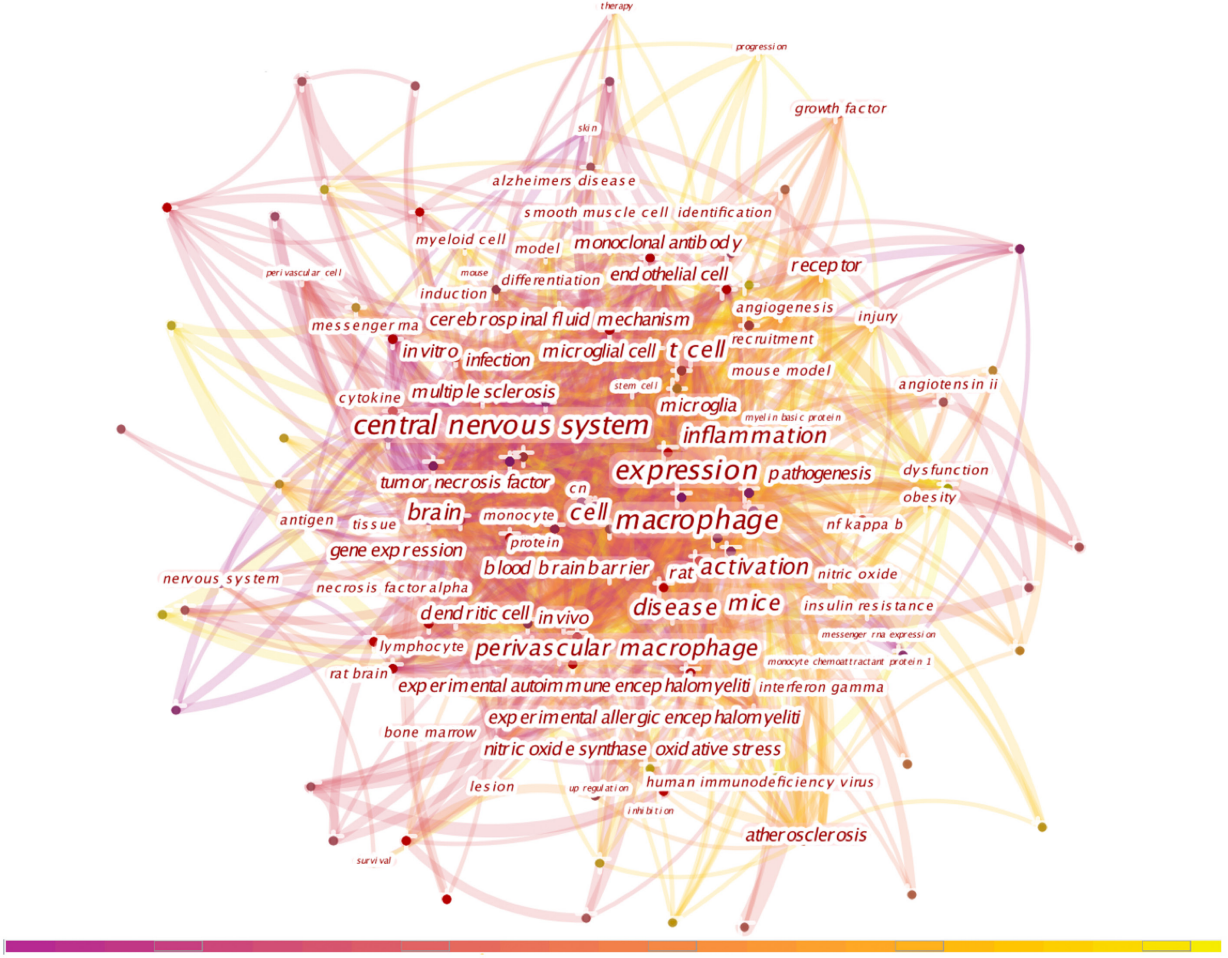
Citespace keyword co‐occurrence analysis network in perivascular macrophages from 1997 to 2021. Each node represents one keyword. The frequently used keywords are exhibited beside the node.

Citespace keyword co‐occurrence analysis network is shown in Figure 3. The top 10 keywords by frequency are shown in Table S2 and they focused on the central nervous system, inflammation, macrophage or T‐cell, disease, and basic research such as expression or activation.

**FIGURE 4:**
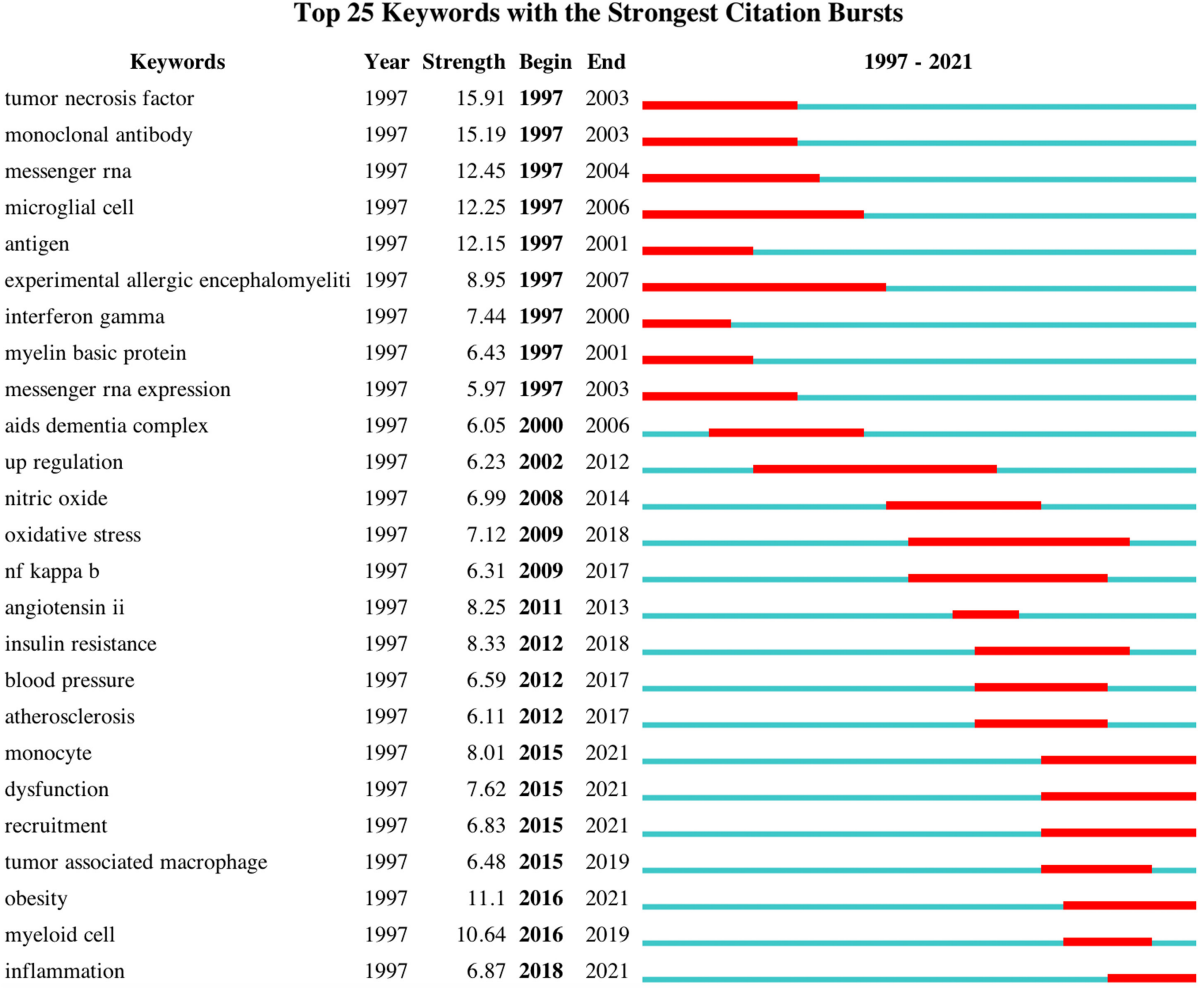
Citespace top 25 keywords burst detection in perivascular macrophages from 1997 to 2021, together with their strength and duration. The strength value reflected the frequency of citation. The green blocks represented the year 1997–2021. The red blocks represented the begin, the end, and the duration of citation bursts.

The top 25 keywords burst detection (Figure 4) found that tumor necrosis factor, monoclonal antibody, messenger RNA, and microglial cell were keywords that emerged earlier and received early attention. Oxidative stress, angiotensin II, and insulin resistance were keywords with high emergence intensity that appeared during the period which might be turning points in the field. Tumor‐associated macrophage, obesity, myeloid cell, and inflammation were relatively recent highlight keywords that attracted attention in recent years. Experimental allergic encephalomyelitis and upregulation were keywords with the longest emergence time.

### 3.4 Institution co‐occurrence analysis highlighted PVM research from Harvard Univ, Vrije Univ Amsterdam

**FIGURE 5:**
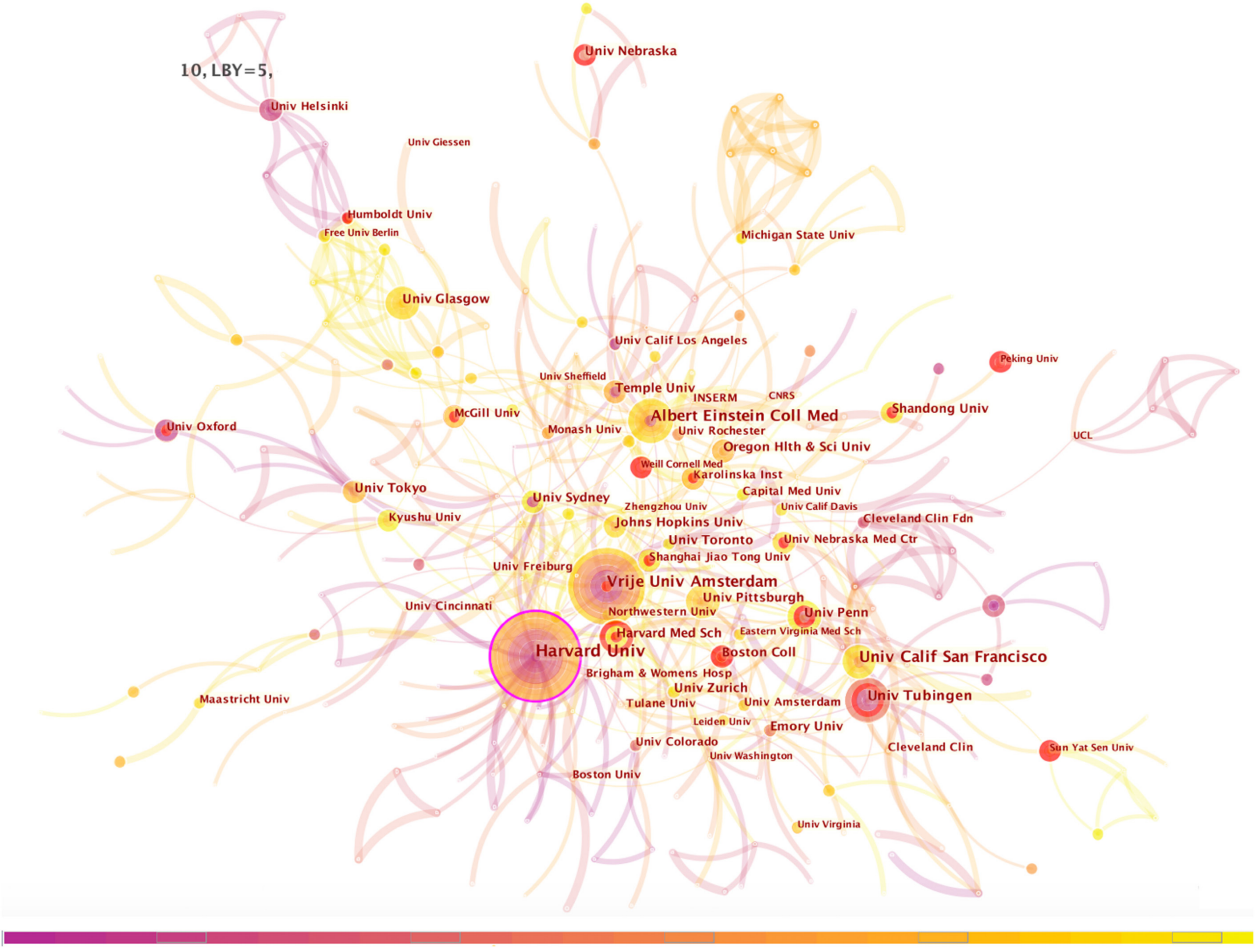
Citespace institution co‐occurrence analysis network in perivascular macrophages from 1997 to 2021. Each node represents one institution and the size of the node reflects its frequency. The institution labels are exhibited beside the node. Centrality is counted and shown as “purple circle” that connect several articles and play a pivot role in the network. The “red circle” means the institution was or is still a hot spot.

Citespace institution co‐occurrence analysis network is shown in Figure 5. The top 10 institutions by frequency, burst, and sigma are shown in Table S3.

Harvard Univ, Vrije Univ Amsterdam, occupied important positions in the field of PVMs. Albert Einstein Coll Med, Univ Calif San Francisco, had “big circle,” but no “purple” or “red” circle. Univ Tubingen, Univ Nebraska, Harvard Med Sch, and Boston Coll were institutions with “red circle” and high burst and sigma values. Peking Univ, Sun Yat Sen Univ, Shanghai Jiao Tong Univ, and Shandong Univ were four universities in China that emerged as “red circle” institutions.

### 3.5 PVM research clusters shown in the network of reference co‐citation analysis

**FIGURE 6:**
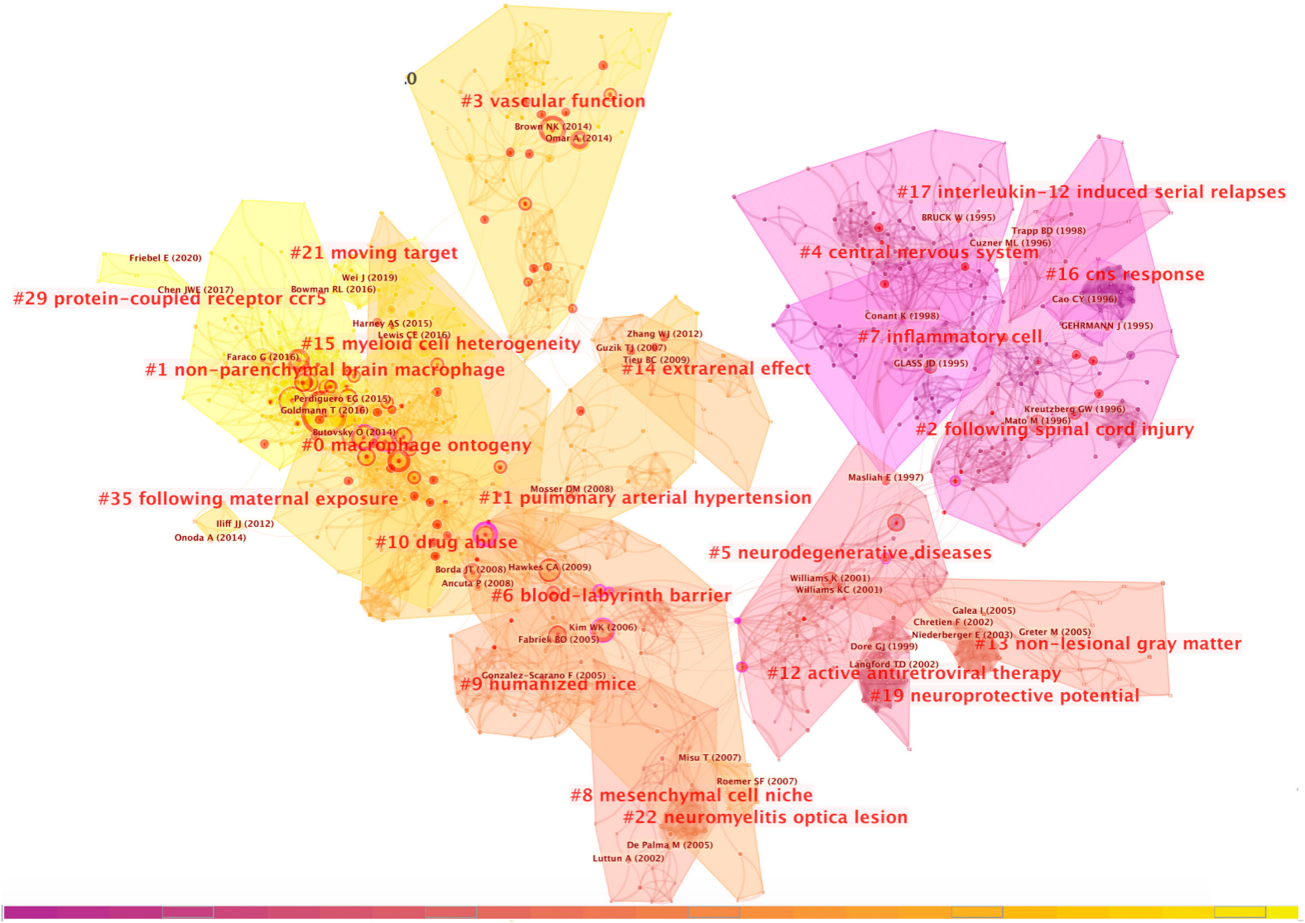
Citespace Reference co‐citation analysis network (Cluster View) in perivascular macrophages from 1997 to 2021. The network has a modularity of 0.8446 and an average silhouette score of 0.9382. The labels of each cluster are exhibited beside the blocks. The color of the clusters shows when the co‐citation links happened. The red color means the citing year is relatively early, and the yellow color indicates that the citation time is relatively recent.

In order to get a cluster view, which provides a very intuitive and accurate look for readers to understand the evolution path of each cluster in the PVMs field, we analyzed the cited references of the retrieved 2384 articles and found that the network has 1329 references and 205 clusters; the main 23 clusters are shown in Figure 6. The top five clusters contain 477 nodes which account for 35.9% in total. The cluster network has modularity of 0.8446 and an average silhouette score of 0.9382. The top 11 references by frequency are listed in Table S4, Cluster #1 has seven references that account for the vast majority. The top one cited reference by frequency in Cluster #1 written by Goldmann T in 2016 discussed the origin, fate, and dynamics of non‐parenchymal macrophages in CNS.^32^ The properties of major clusters are shown in Table S5.

**FIGURE 7:**
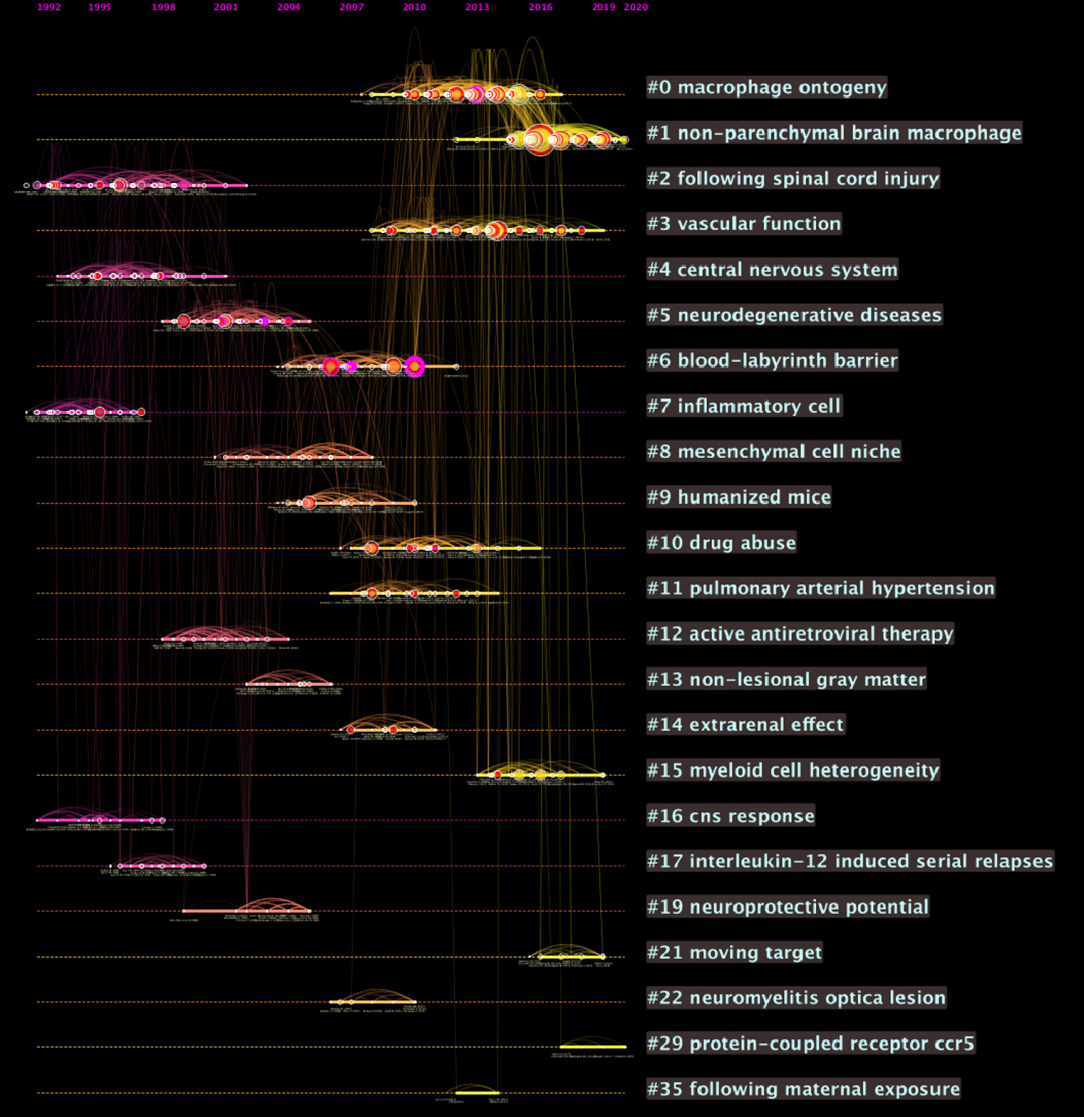
Citespace Reference co‐citation analysis network (Timeline View) in perivascular macrophages from 1997 to 2021. Each node represents one reference. Clusters are listed in descending order according to their size. Cluster labels are exhibited on the right part. The color of the clusters shows when the co‐citation links happened. The red color means the citing year is relatively early, and the yellow color indicates that the citation time is relatively recent. The size of the node represents its frequency, “purple circle” and “red circle” represents its centrality and degree of heat respectively.

In order to get a timeline view of the evolution process and important references or authors in the PVMs field, we placed the cited references in a timeline vision listed in descending order according to their size (Figure 7). Each node represents one reference. The size of the node represents its frequency; “purple circle” and “red circle” represent its centrality and degree of heat, respectively. Cluster #0: macrophage ontogeny, Cluster #1: non‐parenchymal brain macrophage, Cluster #2: following spinal cord injury, Cluster #3: vascular function, Cluster #5: neurodegenerative diseases, and Cluster #6: blood–labyrinth barrier are explained in detail. Cluster #4 is not counted because of its relatively small size and is outdated.

**FIGURE 8:**
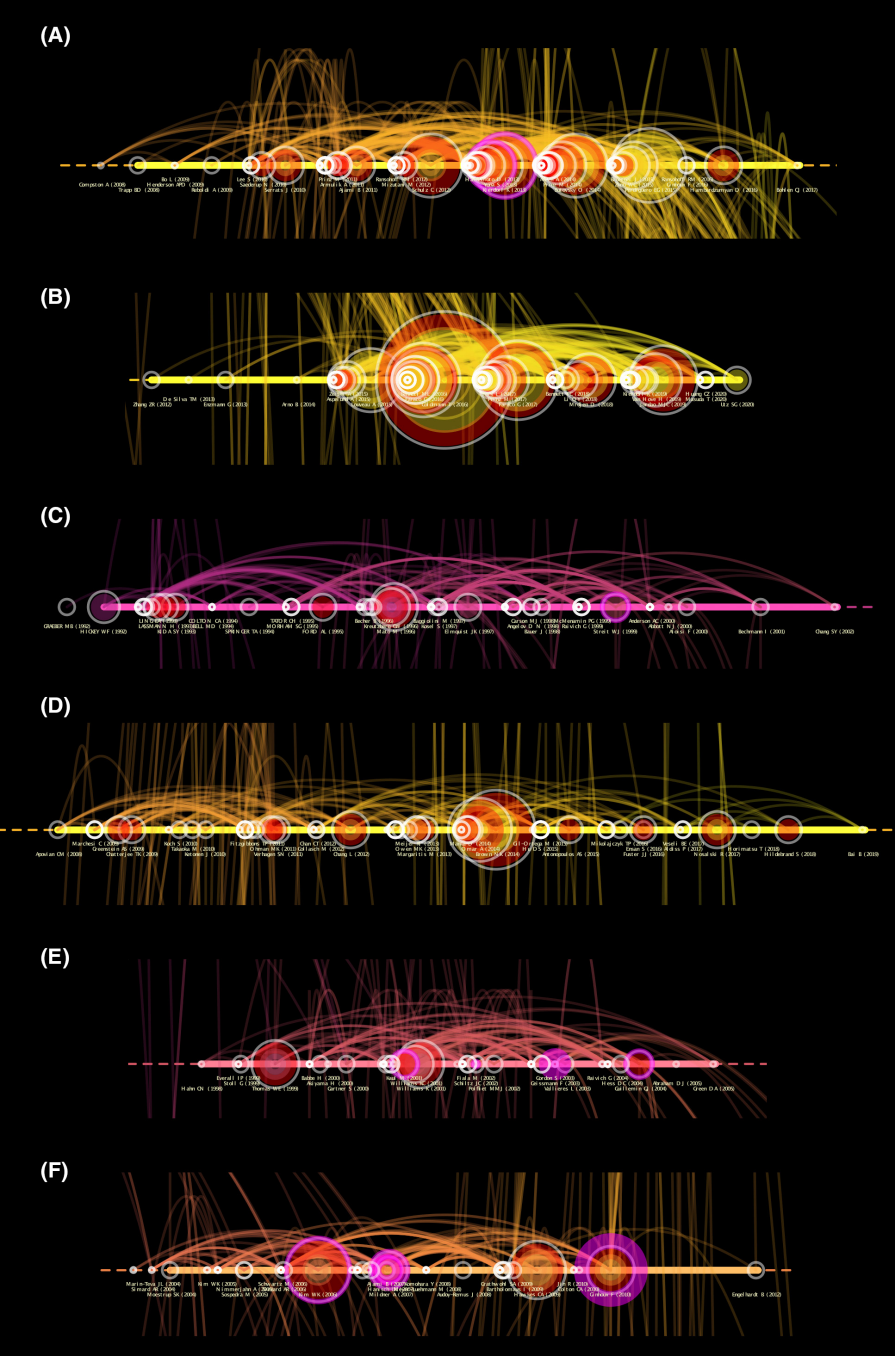
Citespace partial zoom of Cluster #0 (A), #1 (B), #2 (C), #3 (D), #5 (E) and #6 (F) reference co‐citation analysis network (Timeline View) in perivascular macrophages from 1997 to 2021. The label of the most cited reference is placed at the lowest position. The color of the clusters shows when the co‐citation links happened. The red color means the citing year is relatively early, and the yellow color indicates that the citation time is relatively recent. The size of the node represents its frequency, “purple circle” and “red circle” represents its centrality and degree of heat respectively.

Through cluster exploration, we characterized that Cluster #0 (macrophage ontogeny) was the largest cluster, containing 121 references across a 10‐year period from 2008 till 2017 (Figure 8A). The median year of all references in this cluster is 2012, and the median year of the 11 most representative citing articles is 2016. This cluster's silhouette value was 0.879. The label of the most cited reference is placed at the lowest position. Most of the top 10 citing articles in Cluster #0 are reviews, they discussed CNS myeloid cells' roles in various CNS diseases,^33^, ^34^ such as Alzheimer's disease.^35^, ^36^ Microglia and non‐parenchymal macrophages in the brain^24^ and their potential treatment strategy in multiple sclerosis^37^, ^38^ were also mentioned. PVMs in tumors were also a hot topic.^11^ Most of the top 10 cited references in Cluster #0 are basic studies that focused on the origin, development, and maintenance of microglia,^39^ tissue‐resident macrophages,^1^, ^2^, ^3^, ^40^, ^41^ Nr4a1‐dependent Ly6C (low) monocytes,^40^, ^42^ or brain myeloid subtypes,^43^ and their roles during neurodegeneration,^43^ multiple sclerosis, and experimental autoimmune encephalitis (EAE).^44^ PVMs also had dual roles in immune‐to‐brain signaling.^15^ See Table S6.

### 3.6 Detailed cluster view of the reference co‐citation analysis network

Cluster #1 (non‐parenchymal brain macrophage) contains 121 references across a 9‐year period from 2012 to 2020 (Figure 8B). The median year of all references in this cluster is 2017, and the median year of the 11 most representative citing articles is 2019. This cluster's silhouette value is 0.93. The citing articles in Cluster #1 are very new and have relatively rich content. They discussed CNS‐associated macrophages,^45^, ^46^ tumor‐associated microglia/macrophages in glioblastomas,^12^ and CD4(+) T‐cell.^47^ They pointed out that microglia and border‐associated macrophages are separate entities in terms of ontogeny and gene signature,^48^ and microglia or non‐parenchymal brain macrophages both take part in neurodegenerative diseases,^22^ multiple sclerosis,^37^ and cerebrovascular dysfunction such as stroke, cerebral small vessel disease (cSVD), and Alzheimer's disease.^28^, ^49^ The cited references discussed origin, fate, and dynamics of CNS non‐parenchymal macrophages^32^ and myeloid cells.^50^ They revealed the functions of PVMs in hypertension, Alzheimer's disease pathology, or obesity.^51^, ^52^ See Table S7.

Cluster #2 (following spinal cord injury) contains 83 references across a 11‐year period from 1992 to 2002 (Figure 8C). The median year of all references in this cluster is 1996, and the median year of the 10 most representative citing articles is 2019. This cluster's silhouette value is 0.93. Cluster #2 is relatively old, and the top 10 citing articles were published between 1998 and 2001. Virchow–Robin space is a hot topic.^25^, ^26^, ^27^ Perivascular cells are immunoregulatory cells,^23^, ^53^ including brain dendritic cells and macrophages/microglia,^54^, ^55^ that take part in central nervous system inflammation and connect the CNS with the peripheral immune system. COX‐1+ microglia/macrophages participated in focal cerebral infarctions^26^ and spinal cord injury.^56^ Dobrenis K pointed out a transplantation therapy for CNS disease.^57^ Sigma value of Cluster #2 cited references is relatively low and most of them cannot find the corresponding document. Bechmann I found that brain perivascular spaces are under permanent immune surveillance of blood‐borne macrophages.^27^ Perivascular microglia and meningeal macrophages take part in the systemic immune challenge,^58^ while microglia and macrophages function after CNS injury.^59^ See Table S8.

Cluster #3 (vascular function) contains 78 references across a 12‐year period from 2008 to 2019 (Figure 8D). The median year of all references in this cluster is 2013, and the median year of the 11 most representative citing articles is 2015. This cluster's silhouette value is 0.97. Immune cells infiltrate dysfunctional adipose tissue,^60^ and perivascular adipose tissue participates in the inflammation process and affects cardiovascular disorders including atherosclerosis, hypertension, diabetes, obesity,^5^, ^6^, ^7^, ^8^, ^9^ and atrial fibrillation,^10^ which is an important part of PVMs. Basic studies focused on endothelial dysfunction and vascular disease,^61^ and some researchers have found that mTORC2 in perivascular adipose tissue controls vascular function by regulating inflammatory molecule expression.^62^ Angiotensin 1–7 mimetic AVE0991 and angiotensin II type 1 receptor exhibited anti‐atherosclerotic and anti‐inflammatory actions.^63^, ^64^ The content of cited references is similar to citing articles. Perivascular adipose tissue participates in vascular health and disease such as hypertension, atherosclerosis,^6^, ^65^, ^66^, ^67^ diabetes, and obesity.^5^, ^68^, ^69^, ^70^ Perivascular adipose tissue is also capable of linking metabolic signals to inflammation.^71^, ^72^ P‐selectin glycoprotein ligand‐1^67^ and adiponectin regulate endothelial function in human vessels.^73^ See Table S9.

Cluster #5 (neurodegenerative diseases) contains 73 references across an 8‐year period from 1998 to 2005 (Figure 8E). The median year of all references in this cluster is 2001, and the median year of the 10 most representative citing articles is 2004. This cluster's silhouette value is 0.865. Most of the citing articles in “coverage” were about HIV/SIV. Brain mononuclear phagocytes,^74^, ^75^, ^76^ monocytes, and PVMs^29^, ^30^ participate in the pathogenesis of neurodegenerative diseases such as HIV‐1‐associated dementia, Alzheimer's disease, and Parkinson's disease. CD8(+) T‐cells take part in SIV encephalitis.^77^ Proton magnetic resonance spectroscopic imaging is a tool for assessing neuronal function for HIV‐1‐associated dementia.^75^ These citing articles also found CD163(+) blood monocytes/macrophages are a source of brain PVMs^78^ and their roles in brain systemic inflammation.^79^ Cited references of Cluster #5 have a very high average sigma value. Hess DC demonstrated the hematopoietic origin of microglial and perivascular cells in the brain.^80^ Perivascular cells connect the CNS with the peripheral immune system.^23^, ^81^ PVMs,^31^, ^82^, ^83^ perivascular cells,^23^ CD14+/CD16+ peripheral blood‐derived monocytes^84^ participate in HIV/SIV encephalopathy, meanwhile, PVMs and meningeal macrophages (MM) also play vital roles during the early stages of experimental allergic encephalomyelitis (EAE) development.^85^ See Table S10.

Cluster #6 (blood–labyrinth barrier) contains 69 references across a 9‐year period from 2004 to 2012 (Figure 8F). The median year of all references in this cluster is 2007, and the median year of the 10 most representative citing articles is 2010. This cluster's silhouette value is 0.964. Some of the citing articles in Cluster #6 were basic studies. Features of microglia and neuroinflammation^86^, ^87^ were discussed. Chemokine receptor 2 (CCR2)‐expressing myeloid cells^88^ and immune response^89^, ^90^ take place in Alzheimer's disease. Differentiated brain myeloid subtypes,^43^ microglia, inflammatory macrophages,^44^ or M2 macrophages^91^ participated in neurodegeneration multiple sclerosis and experimental autoimmune encephalitis (EAE). Shi XR^92^ and Dai M^93^ focused on the cochlear blood labyrinth barrier. The cited references focused on the multifaceted activities and maintenance of microglia in the normal and pathologic brain,^94^, ^95^, ^96^ and PVMs in Alzheimer's disease.^97^ CCR2(+) monocytes are direct precursors of microglia,^98^ and CD163(+) blood monocytes/macrophages are a source of brain PVMs.^78^ They also discussed effector T‐cell interactions with meningeal vascular structures in nascent autoimmune CNS lesions.^99^ See Table S11.

Cluster #29 (protein‐coupled receptor CCR5) is a very new cluster, so we analyzed its citing references at the same time. They focused on the tumor microenvironment (TME) in glioblastoma and stromal cells which is the latest research directions and potential therapeutic targets. G‐protein‐coupled receptor CCR5 and perivascular niche are recently discussed hotspots.^13^, ^14^

Collectively, the distribution of the research clusters has a difference in time as well as their importance. The content of each cluster is abundant but relatively messy which needs organizing and summarizing. Similarly, there are intersections between clusters.

## 4 DISCUSSION

In the current study, we used bibliometric analysis methods to study PVMs in keywords, references, authors, categories, and institutions. The cluster view and a timeline view of the reference co‐citation analysis network revealed a new perspective on literary interpretation. PVMs in the brain and PVMs in perivascular adipose tissue were two important fields, while adiposity‐related cognitive impairment might be their potential connection.^100^, ^101^

Since the first description as elongated cells in the perivascular space that uptake exogenous trypan blue and horseradish peroxidase was published in 1980,^102^ PVMs, as one of the brain‐resident immune cells, are getting more and more research attention. Although some reviews have summarized the physiological and pathological characteristics of PVMs,^19^, ^51^ some issues remain to be explored such as their roles in immuneregulation, vascular and lymphatic function, their difference and significance with other resident immune cells, and so on. We innovatively applied the bibliometric analysis to visualize the hotspot research of PVMs, which could be helpful to determine more specific research themes and achieve a more comprehensive understanding.

In the current study, 2384 publications under search terms “perivascular macrophages” and “perivascular macrophage” were found and a total citation number of 120,854 of these papers were achieved which indicated that PVMs remain as a research focus of the current study.

In the Citespace category co‐occurrence analysis, several nodes popped out, such as Pathology, Immunology, and Neuroscience. This is consistent with the concept that PVMs might be the bridge connecting peripheral immunity to central immunity.^15^ We further demonstrated that the research hotspots of PVMs gradually shifted from normal physiological function to their function in various disease models and their role in immune regulatory responses. Research directions have also changed gradually, from HIV/SIV encephalopathy to blood–labyrinth barrier, to CNS myeloid cells and PVMs in tumors, and to CNS‐associated macrophages. Prinz M, in particular, had five top citing and cited references in these fields, and had a review published on Annu Rev Immunol in 2021 which summarized the origin of CNS‐associated macrophages and microglia^46^; he was a pioneer in the field and is still active on the front lines. From the results of the Citespace reference co‐citation timeline analysis, the research that focus on PVMs have progressed in timeline and research content as “Neurodegenerative diseases” (Cluster #5, from 1998 till 2005) to “Blood‐labyrinth barrier” (Cluster #6, from 2004 till 2012) to “Macrophage ontogeny” (Cluster #0, from 2008 till 2017) to “Non‐parenchymal brain macrophages” (Cluster #1, from 2012 till 2020). Moreover, “Vascular function” (Cluster #3) is also an important part of PVMs research.

### 4.1 Research in neurological diseases: HIV/SIV neuropathogenesis, neurodegenerative diseases, and central immune inflammation

Kim WK and Fischer‐Smith T are important authors in part of “neurodegenerative diseases” (Cluster #5), and cited references of which have a very high average sigma value. Kim WK explored the role of monocytes and PVMs in HIV/SIV neuropathogenesis in 2005, and distinguished PVMs by macrophage scavenger receptor CD163.^100^ Fischer‐Smith T found an accumulation of PVMs in HIV encephalitis and the role of CD16^+^/PCNA^+^ in mononuclear phagocyte trafficking.^84^ Harvard Univ occupied important positions in the field of PVMs. Kim WK^29^, ^78^ and Williams K^23^, ^103^ are two outstanding researchers at Harvard Univ; they focused on PVMs in HIV/SIV neuropathogenesis.

In terms of central immune control, microglia have always been an area of intense research. The microglial cell is a keyword that developed early in the keywords co‐occurrence analysis; yet, microglial is still a relatively new topic according to the references' co‐citation analysis. The origin, differentiation, and difference and similarities between microglia and PVMs are always the focus of research.^24^, ^37^, ^44^ The word “Microglial cell” showed a steady trend, although fluctuated up or down over a period of time, generally tends to be a research hotspot. Meanwhile, PVMs participated in experimental allergic encephalomyelitis (EAE) or hypertension. Polfliet MMJ,^85^, ^104^ Galea I,^105^ and Faraco G^52^ were representative researchers of Vrije Univ Amsterdam whose research directions were perivascular and meningeal macrophages in EAE or hypertension. EAE had the longest emergence time of keywords burst from 1997 to 2007, meaning researchers continued to pay attention to them, which is also consistent with the result of cluster analysis.^44^, ^85^

### 4.2 Macrophage ontogeny

Prinz M is an interdisciplinary and interepoch researcher, who had five top citing and cited references in Clusters #0, #1, and #6. He reviewed the differentiation of brain myeloid subtypes during neurodegeneration, which was published in Nature Neuroscience in 2011.^43^ Ajami B is another important author in Cluster #6, who identified two distinct subsets of myelomonocytic cells (infiltrating monocytes and microglia) with distinct roles in multiple sclerosis and EAE.^44^ Some cited references of Cluster #6 focused on the origin of microglia, with a top sigma value (28.65) published in SCIENCE by Ginhoux F, who identified that adult microglia derived from primitive myeloid progenitors by fate mapping analysis and in vivo lineage tracing techniques.^94^

### 4.3 Research in perivascular adipose tissue and vascular system

From the results of the Citespace reference co‐citation timeline analysis, the research that focused on PVMs have developed from far to near as “neurodegenerative diseases” (Cluster #5) to “Blood‐labyrinth barrier” (Cluster #6) to “Macrophage ontogeny” (Cluster #0) to “Non‐parenchymal brain macrophages” (Cluster #1). “Vascular function” (Cluster #3) is also an important part of PVMs research. As perivascular cells, the connection and interaction of PVMs with blood vessels have always been a research hotspot. Cardiovascular system and Cardiology ranked No. 5 in the category of co‐occurrence analysis, while “vascular function” (Cluster #3) ranked No. 4 in cluster co‐citation analysis which is a new and large component of the network. Brown NK and Nosalski R were outstanding researchers in the field of perivascular adipose tissue. CIRCULATION, ARTERIOSCL THROM VAS, CIRC RES, and ATHEROSCLEROSIS accounted for the majority of journals in this cluster. Obesity and perivascular adipose tissue have become an important part of the PVMs field. Faraco G had two cited references in “non‐parenchymal brain macrophage” (Cluster #1) that pointed out PVMs as key components of the brain‐resident immune system with broad implications for the pathogenesis of major brain diseases, hypertension, or obesity.^51^, ^52^

### 4.4 Research in the tumor

“An Update on Glioblastoma Biology, Genetics, and Current Therapies: Novel Inhibitors of the G Protein‐Coupled Receptor CCR5” written by Turnsek TL is the representative article. G‐protein‐coupled receptor CCR5 and perivascular niches are research hotspots and directions.

Tumor‐associated macrophage, obesity, myeloid cell, and inflammation were relatively recent highlight keywords that attracted attention in recent years. These words can be considered as one of those future research trends. These are identical to the timeline analysis of the cited articles and citing references.^11^, ^12^, ^13^

Peking Univ, Sun Yat Sen Univ, Shanghai Jiao Tong Univ, and Shandong Univ were four universities in China that emerged as “red circle” institutions meaning Chinese research are on the rise. Their research focused on tumor‐associated macrophages in glioblastoma and perivascular adipose tissue that keep up with the trend of the times. More and more authors and research institutes are paying attention to this hotspot, indicating that this field has yet to be explored.

In general, HIV/SIV encephalopathy (Cluster #5, from 1998 to 2005) and Virchow–Robin space (Cluster #2, from1992 to 2002) have been well‐addressed. CNS‐associated macrophages (Cluster #1, from 2012 to 2020), perivascular adipose tissue (Cluster #3, from 2008 to 2019), and tumor microenvironment (Cluster #29, from 2017 till now) are the latest research directions and potential therapeutic targets for cerebrovascular or cardiovascular diseases. The category and origin of CNS‐associated macrophages still remain controversial and need to be addressed. Back to Citespace keywords burst detection, it is of great practical value. Readers who do not have time to browse the full text of every article in one field can discover key research through keyword burst detection. This method detects rapidly growing specialized vocabulary in a short period of time through the statistics of citation keywords. However, whether these burst keywords are truly worth studying with significant translational value needs to be further considered in conjunction with cluster analysis, which can show the structure and timeline of one field by clustering. Combined keywords burst detection and cluster analysis, we suggest “tumor associated macrophage,” “obesity,” and “inflammation” as “important” and “hot” in‐depth research directions.

The function and role of PVMs in various disease models have been explored in recent years. Proinflammatory signaling modulating blood–brain barrier function and tau pathology, or peripherally derived PVMs targeting amyloid plaques have been devoted to Alzheimer's disease.^104^, ^105^, ^106^ Col1a1 expressed by PVMs supports the concept that PVMs are involved in hypertension.^107^ IDO‐1 expression in PVMs might suppress the overactivation of inflammatory reactions.^108^ PVMs even take part in neurogenic inflammation responses in psychiatric disorders.^109^ A frontier study has also reviewed the PVMs' contribution to the development of obesity‐related CNS inflammation and neuroendocrine control.^110^ Although these convincing preclinical evidences suggest PVMs as a promising target for many diseases, there are many difficulties in translating these results into clinical application,^111^ and the understanding of these special cells is yet to be deepened. PVMs are perivascular cells, but their interaction with CD8+ T‐cells^112^ or regulation of blood vessels, especially damaged blood vessels such as stroke, brain hemorrhage, blood–brain barrier damage, or angiogenesis remain poorly understood.^104^ The growth and migration of tumors depend on blood vessels, and the function of PVMs in this regard is still unknown. Meanwhile, as an inherent phagocytic cell in the brain, PVMs were considered different cell types from microglia in terms of origin and differentiation. But their synergy, interaction, and difference with microglia remain to be explored. From the perspective of phenotype, with the development of single‐cell technology, researchers have demonstrated that PVMs exhibited characteristic phenotypes such as CD163, CD169, and LYVE1,^113^ and novel markers such as c‐MAF,^114^ ANXA3,^115^ etc. However, it remains unclear what these phenotypes represent and what functions they play in physiological and pathological conditions, which may represent as possible future directions for further research. Moreover, only a few studies have explored the nature of PVMs, therefore, defining cell‐type‐specific functions of PVMs and establishing humanized model systems of CNS diseases or cerebrovascular diseases and tumors are of great biological significance. According to the existing studies on PVM in pathological diseases, the role of chemokines, cytokines, and their ligands seem to have biological potential, such as CCL2 in neurodegenerative diseases,^46^ IDO‐1 in neuroinflammatory,^108^ CCR5 in TME,^13^ or IL‐6 and TNF‐α in cardiovascular disorders.^5^

This study has some limitations: (1) Some authors did not put important words that are present in the title as “keywords,” which might have a sort of bias in the keyword analysis. (2) The search method and search terms can be optimized.

In conclusion, we emphasize the importance of bibliometric analysis through a detailed interpretation of Citespace cluster and timeline analysis in order to obtain the relationship between important clusters and outstanding authors that devoted themselves to a certain cluster or continue to deepen their research between clusters over time. The current PVMs bibliometric analysis can make a more intuitive understanding of PVMs field in terms of space, time, and authors relationship, thus not only bringing hope to the novel treatment targets for brain or peripheral vascular diseases but also providing insights into the research of obesity and many other major organ diseases.

## CONFLICT OF INTEREST

Dr. Peiying Li is an Editorial Board member of CNS Neuroscience and Therapeutics and a co‐author of this article. To minimize bias, they were excluded from all editorial decision‐making related to the acceptance of this article for publication.

## Supporting information


TableS1‐S11

## Data Availability

The original contributions presented in the study are included in the article/supplementary material, and further inquiries are available by contacting the journal editor or corresponding/first authors.
