# Supplementary material for: Trends in perivascular macrophages research from 1997 to 2021: A bibliometric analysis
Source: CNS Neurosci Ther. 2022 Dec 13;29(3):816–30. doi: 10.1111/cns.14034 (PMC9928555; doi:10.1111/cns.14034)
Supplement: Supplementary file 1 — TableS1‐S11 [file CNS-29-816-s001.docx]

Supplementary Tables:

Supplementary Table 1. Top 10 Categories by Frequency, Burst and Sigma in Perivascular Macrophages from 1997 to 2021.

| Freq | Category | Burst | Category | Sigma | Category |
| --- | --- | --- | --- | --- | --- |
| 644 | Neurosciences & Neurology | 18.97 | Pathology | 8.91 | Pathology |
| 578 | Neurosciences | 11.70 | Chemistry | 3.05 | Science & Technology-Other Topics |
| 331 | Immunology | 11.22 | Chemistry, Multidisciplinary | 2.72 | Pharmacology & Pharmacy |
| 324 | Pathology | 9.80 | Multidisciplinary Sciences | 2.54 | Surgery |
| 267 | Cardiovascular system & Cardiology | 8.68 | Science & Technology-Other Topics | 2.25 | Immunology |
| 260 | Clinical Neurology | 7.02 | Surgery | 1.33 | Research & Experimental Medicine |
| 190 | Peripheral Vascular Disease | 6.30 | Research & Experimental Medicine | 1.33 | Medicine, Research & Experimental |
| 182 | Cell Biology | 6.30 | Medicine, Research & Experimental | 1.28 | Materials Science |
| 145 | Cardiac & Cardiovascular Systems | 5.31 | Cell & Tissue Engineering | 1.27 | Chemistry |
| 143 | Biochemistry & Molecular Biology | 5.21 | Pharmacology & Pharmacy | 1.26 | Chemistry, Multidisciplinary |

Supplementary Table 2. Top 10 Keywords by Frequency in Perivascular Macrophages from 1997 to 2021.

| Freq | Keyword |
| --- | --- |
| 400 | Expression |
| 359 | Central Nervous System |
| 246 | Macrophage |
| 241 | Cell |
| 186 | Inflammation |
| 172 | Activation |
| 133 | T Cell |
| 133 | Disease |
| 128 | Brain |
| 125 | Mice |

Supplementary Table 3. Top 11 Institutions by Frequency, Burst and Sigma in Perivascular Macrophages from 1997 to 2021.

| Freq | Institution | Burst | Institution | Sigma | Institution |
| --- | --- | --- | --- | --- | --- |
| 45 | Harvard Univ | 7.46 | Univ Tubingen | 1.39 | Univ Penn |
| 33 | Vrije Univ Amsterdam | 5.92 | Univ Nebraska | 1.30 | Harvard Med Sch |
| 32 | Albert Einstein Coll Med | 5.87 | Univ Glasgow | 1.26 | Vrije Univ Amsterdam |
| 26 | Univ Calif San Francisco | 5.53 | Harvard Med Sch | 1.21 | Cleveland Clin Fdn |
| 23 | Univ Tubingen | 5.37 | Boston Coll | 1.18 | Monash Univ |
| 16 | Oregon Hlth & Sci Univ | 5.02 | Oregon Hlth & Sci Univ | 1.14 | Karolinska Inst |
| 16 | Temple Univ | 4.90 | Univ Helsinki | 1.11 | Univ Tubingen |
|  |  | 4.50 | Cleveland Clin Fdn | 1.10 | Univ Glasgow |
|  |  | 4.19 | Vrije Univ Amsterdam | 1.07 | Oregon Hlth & Sci Univ |
|  |  | 4.14 | Univ Oxford | 1.07 | Boston Coll |
|  |  |  |  | 1.07 | INSERM |

Supplementary Table 4. Top 11 References by Frequency in Perivascular Macrophages from 1997 to 2021.

| Top 11 References by Frequency | | |
| --- | --- | --- |
| Freq | Author (Year), Journal, Volume, Page | Cluster |
| 70 | Goldmann T (2016), Nat Immunol, 17, 797 | 1 |
| 32 | Faraco G (2016), J Clin Invest, 126, 4674 | 1 |
| 31 | Bennett ML (2016), P Natl Acad Sci USA, 113, 0 | 1 |
| 30 | Brown NK (2014), Arterioscl Throm Vas, 34, 1621 | 3 |
| 29 | Faraco G (2017), J Mol Med, 95, 1143 | 1 |
| 26 | Kim WK (2006), AM J Pathol, 168, 822 | 6 |
| 26 | Butovsky O (2014), Nat Neurosic, 17, 131 | 0 |
| 24 | Louveau A (2015), Nature, 523, 337 | 1 |
| 24 | Perdiguero EG (2015), Nature, 518, 547 | 0 |
| 21 | Prinz M (2017), Nat Immunol, 18, 385 | 1 |
| 21 | Jordao MJC (2019), Science, 363, 365 | 1 |

Supplementary Table 5. Properties of Major Clusters in Perivascular Macrophages from 1997 to 2021.

| Cluster ID | Size | Silhouette | From | To | Mean | Duration | Theme |
| --- | --- | --- | --- | --- | --- | --- | --- |
| 0 | 121 | 0.879 | 2008 | 2017 | 2012 | 10 | macrophage ontogeny |
| 1 | 121 | 0.93 | 2012 | 2020 | 2017 | 9 | non-parenchymal brain macrophage |
| 2 | 83 | 0.93 | 1992 | 2002 | 1996 | 11 | following spinal cord injury |
| 3 | 78 | 0.97 | 2008 | 2019 | 2013 | 12 | vascular function |
| 4 | 74 | 0.913 | 1993 | 2001 | 1996 | 9 | neuroinflammatory products |
| 5 | 73 | 0.865 | 1998 | 2005 | 2001 | 8 | neurodegenerative diseases |
| 6 | 69 | 0.964 | 2004 | 2012 | 2007 | 9 | blood-labyrinth barrier |

Supplementary Table 6. Top 11 Coverage and Top 10 Sigma of Cluster #0 in Perivascular Macrophages from 1997 to 2021.

| Citing Articles | | Cited References | |
| --- | --- | --- | --- |
| Coverage | Author, Year, Title | Sigma | Author, Year, Source, Vol, Page |
| 27 | Prinz, M (2017) Ontogeny and homeostasis of CNS myeloid cells | 2.41 | Kierdorf K, 2013, NAT NEUROSCI, 16, 273 |
| 20 | Herz, J (2017) Myeloid Cells in the Central Nervous System | 1.89 | Hashimoto D, 2013, IMMUNITY, 38, 792 |
| 19 | Lopez-Atalaya, JP (2018) Development and maintenance of the brain's immune toolkit: Microglia and non-parenchymal brain macrophages | 1.52 | Schulz C, 2012, SCIENCE, 336, 86 |
| 19 | Wang, JY (2019) Targeting Microglia and Macrophages: A Potential Treatment Strategy for Multiple Sclerosis | 1.49 | Carlin LM, 2013, CELL, 153, 362 |
| 18 | Koeniger, T (2017) Splitting the “Unsplittable”: Dissecting Resident and Infiltrating Macrophages in Experimental Autoimmune Encephalomyelitis | 1.40 | Ajami B, 2011, NAT NEUROSCI, 14, 1142 |
| 16 | Konishi H (2017) Siglec-H is a microglia-specific marker that discriminates microglia from CNS-associated macrophages and CNS-infiltrating monocytes | 1.18 | Perdiguero EG, 2015, NATURE, 518, 547 |
| 16 | Bogie, JF (2014) Macrophage subsets and microglia in multiple sclerosis | 1.18 | Bruttger J, 2015, IMMUNITY, 43, 92 |
| 14 | Meyer-Luehmann, M (2015) Myeloid Cells in Alzheimer’s Disease: Culprits, Victims or Innocent Bystanders? | 1.17 | Yona S, 2013, IMMUNITY, 38, 79 |
| 13 | Wong, K (2017) Mice deficient in NRROS show abnormal microglial development and neurological disorders | 1.15 | Prinz M, 2011, NAT NEUROSCI, 14,1227 |
| 13 | Brendecke, SM (2015) Do not judge a cell by its cover—diversity of CNS resident, adjoining and infiltrating myeloid cells in inflammation | 1.13 | Serrats J, 2010, NEURON, 65, 94 |
| 13 | Kiss, M (2018) Myeloid cell heterogeneity in cancer: not a single cell alike |  |  |

Supplementary Table 7. Top 11 Coverage and Top 10 Sigma of Cluster #1 in Perivascular Macrophages from 1997 to 2021.

| Citing Articles | | Cited References | |
| --- | --- | --- | --- |
| Coverage | Author, Year, Title | Sigma | Author, Year, Source, Vol, Page |
| 30 | Prinz M (2021) Central Nervous System-Associated Macrophages-From Origin to Disease Modulation | 3.97 | Goldmann T, 2016, NAT IMMUNOL, 17, 797 |
| 24 | Kierdorf K (2019) Macrophages at CNS interfaces: ontogeny and function in health and disease | 1.45 | Buttgereit A, 2016, NAT IMMUNOL, 17, 1397 |
| 21 | Lee E (2021) Distinct Features of Brain-Resident Macrophages: Microglia and Non-Parenchymal Brain Macrophages | 1.21 | Faraco G, 2016, J CLIN INVEST, 126, 4674 |
| 17 | Koizumi T (2019) Vessel-Associated Immune Cells in Cerebrovascular Diseases: From Perivascular Macrophages to Vessel-Associated Microglia | 1.17 | Louveau A, 2015, NATURE, 523, 337 |
| 16 | Pires-Afonso Y (2020) Revealing and Harnessing Tumour-Associated Microglia/Macrophage Heterogeneity in Glioblastoma | 1.12 | Prinz M, 2017, NAT IMMUNOL, 18, 385 |
| 16 | Masuda T (2020) Novel Hexb-based tools for studying microglia in the CNS | 1.11 | Jordao MJC, 2019, SCIENCE, 363, 365 |
| 15 | Wang JY (2019) Targeting Microglia and Macrophages: A Potential Treatment Strategy for Multiple Sclerosis | 1.10 | Faraco G, 2017, J MOL MED, 95, 1143 |
| 14 | Li Y (2020) Microglia, autonomic nervous system, immunity and hypertension: Is there a link? | 1.10 | Bennett ML, 2016, P NATL ACAD SCI USA, 113, 0 |
| 14 | Mundt, S (2019) The CNS Immune Landscape from the Viewpoint of a T Cell | 1.07 | Zeisel A, 2015, SCIENCE, 347, 1138 |
| 13 | Ross JM (2020) The Expanding Cell Diversity of the Brain Vasculature | 1.07 | Aspelund A, 2015, J EXP MED, 212, 991 |
| 13 | Utz SG (2020) Early Fate Defines Microglia and Non-parenchymal Brain Macrophage Development |  |  |

Supplementary Table 8. Top 10 Coverage and Sigma of Cluster #2 in Perivascular Macrophages from 1997 to 2021.

| Citing Articles | | Cited References | |
| --- | --- | --- | --- |
| Coverage | Author, Year, Title | Sigma | Author, Year, Source, Vol, Page |
| 14 | Hickey WF (2001) Basic principles of immunological surveillance of the normal central nervous system | 3.12 | Streit WJ, 1999, PROG NEUROBIOL, 57, 563 |
| 12 | Matyszak MK (1998) Inflammation in the CNS: Balance between immunological privilege and immune responses | 1.84 | Kreutzberg GW, 1996, TRENDS NEUROSCI, 19, 312 |
| 12 | Bechmann I (2001) Immune surveillance of mouse brain perivascular spaces by blood-borne macrophages | 1.09 | Elmquist JK, 1997, J COMP NEUROL, 381, 119 |
| 12 | Schwab JM (2000) Selective accumulation of cyclooxygenase-1-expressing microglial cells/macrophages in lesions of human focal cerebral ischemia | 1.07 | Bechmann I, 2001, EXP NEUROL, 168, 242 |
| 12 | Schwab JM (2000) Persistent accumulation of cyclooxygenase-1 (COX-1) expressing microglia/macrophages and upregulation by endothelium following spinal cord injury | 1.05 | KIDA SY, 1993, ACTA NEUROPATHOL, 85,646 |
| 10 | Dobrenis K (1998) Microglia in cell culture and in transplantation therapy for central nervous system disease | 1.03 | Angelov DN, 1996, GLIA, 16, 129 |
| 10 | Fischer HG (2001) Brain dendritic cells and macrophages/microglia in central nervous system inflammation | 1.01 | FORD AL, 1995, J IMMUNOL, 154, 4309 |
| 10 | Bechmann I (2001) Turnover of rat brain perivascular cells | 1.01 | Becher B, 1996, GLIA, 18, 1 |
| 8 | Perry VH (1998) A reised view of the central nervous system microenvironment and major histocompatibility complex class ii antigen presentation | 1.01 | LASSMANN H, 1993, GLIA, 7, 19 |
| 7 | Williams K (2001) Central nervous system perivascular cells are immunoregulatory cells that connect the CNS with the peripheral immune system | 1.01 | Mato M, 1996, P NATL ACAD SCI USA, 93, 3269 |

Supplementary Table 9. Top 11 Coverage and Sigma of Cluster #3 in Perivascular Macrophages from 1997 to 2021.

| Citing Articles | | Cited References | |
| --- | --- | --- | --- |
| Coverage | Author, Year, Title | Sigma | Author, Year, Source, Vol, Page |
| 21 | Nosalski R (2017) Perivascular adipose tissue inflammation in vascular disease | 1.85 | Brown NK, 2014, ARTERIOSCL THROM VAS, 34,1621 |
| 12 | Guzik TJ (2017) The role of infiltrating immune cells in dysfunctional adipose tissue | 1.20 | Fuster JJ, 2016, CIRC RES, 118, 1786 |
| 11 | Brown NK (2014) Perivascular Adipose Tissue in Vascular Function and Disease A Review of Current Research and Animal Models | 1.16 | Chang L, 2012, CIRCULATION, 126, 1067 |
| 10 | Srikakulapu P (2017) Perivascular Adipose Tissue Harbors Atheroprotective IgM-Producing B Cells | 1.13 | Ohman MK, 2011, ATHEROSCLEROSIS, 219, 33 |
| 10 | Gu P (2013) Interplay between adipose tissue and blood vessels in obesity and vascular dysfunction | 1.10 | Nosalski R, 2017, BRIT J PHARMACOL, 174, 3496 |
| 10 | Van de Voorde J (2014) Perivascular Adipose Tissue, inflammation and vascular dysfunction in obesity | 1.06 | Omar A, 2014, ARTERIOSCL THROM VAS, 34, 1631 |
| 9 | Chistiakov DA (2017) Impact of the cardiovascular system-associated adipose tissue on atherosclerotic pathology | 1.06 | Verhagen SN, 2011, ATHEROSCLEROSIS, 214, 3 |
| 9 | Bhattacharya I (2013) Rictor in Perivascular Adipose Tissue Controls Vascular Function by Regulating Inflammatory Molecule Expression | 1.04 | Margaritis M, 2013, CIRCULATION, 127, 2209 |
| 8 | Skiba DS (2017) Anti-atherosclerotic effect of the angiotensin 1-7 mimetic AVE0991 is mediated by inhibition of perivascular and plaque inflammation in early atherosclerosis | 1.03 | Chatterjee TK, 2009, CIRC RES, 104, 541 |
| 7 | Irie D (2015) Transplantation of periaortic adipose tissue from angiotensin receptor blocker-treated mice markedly ameliorates atherosclerosis development in apoE(-/-) mice | 1.02 | Greenstein AS, 2009, CIRCULATION, 119, 1661 |
| 7 | Vanhoutte PM (2017) Endothelial dysfunction and vascular disease - a 30th anniversary update | 1.02 | Antonopoulos AS, 2015, DIABETES, 64, 2207 |

Supplementary Table 10. Top 10 Coverage and Top 9 Sigma of Cluster #5 in Perivascular Macrophages from 1997 to 2021.

| Coverage | Author, Year, Title | Sigma | Author, Year, Source, Vol, Page |
| --- | --- | --- | --- |
| Citing Articles | | Cited References | |
| 12 | Kadiu I (2005) Mononuclear phagocytes in the pathogenesis of neurodegenerative diseases | 2.82 | Kaul M, 2001, NATURE, 410,988 |
| 11 | Kim WK (2005) The role of monocytes and perivascular macrophages in HIV and SIV neuropathogenesis: Information from non-human primate models | 2.42 | Guillemin GJ, 2004, J LEUKOCYTE BIOL, 75, 388 |
| 9 | Kim WK (2006) CD163 identifies perivascular macrophages in normal and viral encephalitic brains and potential precursors to perivascular macrophages in blood | 2.40 | Williams K, 2001, GLIA, 36, 156 |
| 9 | Everall IP (2005) The shifting patterns of HIV encephalitis neuropathology | 1.83 | Williams KC, 2001, J EXP MED, 193, 905 |
| 8 | Nelson JA (2005) Coregistration of quantitative proton magnetic resonance spectroscopic Imaging with neuropathological and neurophysiological analyses defines the extent of neuronal impairments in murine human immunodeficiency virus type-1 encephalitis | 1.28 | Fischer-Smith T, 2001, J NEUROVIROL, 7, 528 |
| 8 | Nelson JA (2005) Coregistration of quantitative proton magnetic resonance spectroscopic Imaging with neuropathological and neurophysiological analyses defines the extent of neuronal impairments in murine human immunodeficiency virus type-1 encephalitis | 1.14 | Gartner S, 2000, SCIENCE, 287, 602 |
| 7 | Schiltz JC (2003) Signaling the brain in systemic inflammation: The role of perivascular cells | 1.09 | Thomas WE, 1999, BRAIN RES REV, 31, 42 |
| 6 | Fischer-Smith T (2004) Macrophage/microglial accumulation and proliferating cell nuclear antigen expression in the central nervous system in human immunodeficiency virus encephalopathy | 1.05 | Polfliet MMJ, 2002, J NEUROIMMUNOL, 122, 1 |
| 6 | Goehler LE (2006) Neural-immune interface in the rat area postrema | 1.01 | Hess DC, 2004, EXP NEUROL, 186, 134 |
| 5 | Kim WK (2004) Identification of T lymphocytes in simian immunodeficiency virus encephalitis: Distribution of CD8(+) T cells in association with central nervous system vessels and virus |  |  |

Supplementary Table 11. Top 10 Coverage and Top 7 Sigma of Cluster #6 in Perivascular Macrophages from 1997 to 2021.

| Coverage | Author, Year, Title | Sigma | Author, Year, Source, Vol, Page |
| --- | --- | --- | --- |
| Citing Articles | | Cited References | |
| 14 | Kraft AD (2011) Features of Microglia and Neuroinflammation Relevant to Environmental Exposure and Neurotoxicity | 28.65 | Ginhoux F, 2010, SCIENCE, 330, 841 |
| 13 | Davoust N (2008) From bone marrow to microglia: barriers and avenues | 5.65 | Kim WK, 2006, AM J PATHOL, 168, 822 |
| 12 | Prinz M (2011) Heterogeneity of CNS myeloid cells and their roles in neurodegeneration | 2.74 | Mildner A, 2007, NAT NEUROSCI, 10, 1544 |
| 10 | Nichol KE (2008) Exercise alters the immune profile in Tg2576 Alzheimer mice toward a response coincident with improved cognitive performance and decreased amyloid | 2.71 | Ajami B, 2007, NAT NEUROSCI, 10, 1538 |
| 9 | Mildner A (2011) Distinct and Non-Redundant Roles of Microglia and Myeloid Subsets in Mouse Models of Alzheimer's Disease | 1.26 | Hawkes CA, 2009, P NATL ACAD SCI USA, 106, 1261 |
| 8 | Shi XR (2010) Resident macrophages in the cochlear blood-labyrinth barrier and their renewal via migration of bone-marrow-derived cells | 1.14 | Bartholomaus I, 2009, NATURE, 462, 94 |
| 7 | Lourbopoulos A (2011) Administration of 2-arachidonoylglycerol ameliorates both acute and chronic experimental autoimmune encephalomyelitis | 1.13 | Hanisch UK, 2007, NAT NEUROSCI, 10, 1387 |
| 6 | Dai M (2010) Bone Marrow Cell Recruitment Mediated by Inducible Nitric Oxide Synthase/Stromal Cell-Derived Factor-1 alpha Signaling Repairs the Acoustically Damaged Cochlear Blood-Labyrinth Barrier |  |  |
| 6 | Wilcock DM (2011) Diverse inflammatory responses in transgenic mouse models of Alzheimer's disease and the effect of immunotherapy on these responses |  |  |
| 6 | Ajami B (2011) Infiltrating monocytes trigger EAE progression, but do not contribute to the resident microglia pool |  |  |
